# 

**Published:** 1895-07-27

**Authors:** 


### CONTENTS.

The British Medical Association in London - - - 281

The Misuse of Hospitals. III.—Points for the Proposed Conference - - - 282

Modern Medico-Psychology and Psychiatry. By T. S. Clouston, M.D. - - - 283

Medical Progress and Hospital Clinics—

On the Localising of Fractures of the Base of the Skull - - - 287

Progress in Medicine.—Disease of the Respiratory System (continued) - - - 288

Progress in Surgery.—Cerebral Surgery (continued) - - - 289

Progress in Ophthalmology - - - 291

New Appliances and Things Medical - - - 292

Beef-Tea: Its Body and Soul - - - 284

Annotations.—Eucalyptus Treatment at Leicester—The Poplar Hospital in Danger—"Self Help" and the "Heads of the Profession" - - - 285

Notes and News - - - 286

The Institutional Workshop—

Hospital Construction - - - 293

Hospital Sunday Fund - - - 294

Practical Departments - - - 294

Hospital Festivals and Meetings - - - 295

Around the Hospitals - - - 296

Scraps and Gleanings - - - 297

### EXTRA SUPPLEMENT.—THE NURSING MIRROR.

News from the Nursing World—"Our Hospital"—At the Great Northern—Grateful Acknowledgments—The First and Second Thousand—A New Ladies' League—Hampstead District Nursing—Women Doctors—Fancy Fees—Society of Trained Masseuses—Combined Day and Night Duty—Nurses for Country Villages—Medals for French Attendants—Pinafores and Politics—The Yarrow Home—Queen's Nurses in Scotland—The Royal British Nurses Association—Short Items cix

Elementary Anatomy and Surgery for Nurses. By W. McAdam Eccles, M.D., M.S., F.R.C.S. XXVI.—The Special Senses (continued) - - - cxi

National Health Society - - - cxi

Nursing in Japan.—University Hospital, Tokio - - - cxii

Notes and Queries - - - cxii

Royal National Pension Fund for Nurses - - - cxiii

Registration of Midwives—Friend, Not Busybody - cxiii

Novelties for Nurses - - - cxiv

Holidays and Health - - - cxv

French Schools for Trained Nurses: Their Origin and Organisation - - - cxv

Flat Foot Occurring in Nurses - - - cxvi

For Reading to the Sick - - - cxvi

Everybody's Opinion - - - cxvii

Care of the Sick in Alexandria and Cairo - - - cxvii

